# Lysine-Specific Demethylase 1 (LSD1/KDM1A) Inhibition as a Target for Disease Modification in Myelofibrosis

**DOI:** 10.3390/cells11132107

**Published:** 2022-07-03

**Authors:** Harinder Gill

**Affiliations:** Department of Medicine, LKS Faculty of Medicine, School of Clinical Medicine, The University of Hong Kong, Hong Kong SAR, China; gillhsh@hku.hk; Tel.: +852-22554542

**Keywords:** myelofibrosis, disease modification, LSD1, bomedemstat

## Abstract

Myelofibrosis (MF) is the most symptomatic form of myeloproliferative neoplasm and carries the worst outcome. Allogeneic hematopoietic stem cell transplantation is the only therapy with potential for cure at present, but is limited by significant mortality and morbidity. JAK inhibition is the mainstay of treatment for intermediate- and high-risk MF. Ruxolitinib is the most widely used JAK1/2 inhibitor and provides durable effects in controlling symptom burden and spleen volumes. Nevertheless, ruxolitinib may not adequately address the underlying disease biology. Its effects on mutant allele burden, bone marrow fibrosis, and the prevention of leukemic transformation are minimal. Multiple small molecules are being tested in multiple phase 2 and 3 studies as either monotherapy or in combination with JAK2 inhibitors. In this review, the role of LSD1/KDM1A inhibition as a potential disease-modification strategy in patients with myelofibrosis is described and discussed.

## 1. Introduction

Myeloproliferative neoplasms (MPNs) are unique hematopoietic stem cell disorders sharing mutations that constitutively activate the signal-transduction pathways involved in hematopoiesis [1]. They are characterized by stem cell-derived clonal myeloproliferation. The classical Philadelphia (Ph) chromosome-negative MPNs comprise polycythemia vera (PV), essential thrombocythemia (ET), and primary myelofibrosis (PMF), and are associated with the driver genes *JAK2*, *CALR*, and *MPL*. PMF is characterized by reticulin fibrosis, abnormal cytokine-mediated systemic symptoms, anemia, hepatosplenomegaly, and a propensity for progression to AML. Patients with PV and ET may progress to post-PV (PPV) and post-ET (PET)—myelofibrosis (MF), respectively. The incidence of PV, ET, and PMF is approximately 0.4 to 2.8, 0.38 to 1.7, and 0.1 to 1.0 per 100,000 persons per year, respectively [2]. MPNs generally affect the middle-aged, with the median age at presentation of PV, ET, and PMF being 65, 68, and 70 years, respectively [3]. Patients with MF carry the worst prognosis [4,5]. Median survival for PV, ET, and PMF is 14, 20, and 5.7 years respectively [6,7,8]. Allogeneic hematopoietic stem cell transplantation is the only therapy with potential for cure at present, but is limited by significant mortality and morbidity [9]. Thus, JAK inhibition is the cornerstone of treatment for intermediate- and high-risk MF. Ruxolitinib is the most widely used JAK1/2 inhibitor, provides durable effects in controlling patient symptoms and spleen volumes, and may prolong survival [10,11,12,13,14,15,16]. Nevertheless, the effect of ruxolitinib on mutant allele burden, bone marrow fibrosis, and the prevention of leukemic transformation has been little observed. Even the three other JAK2 inhibitors—fedratinib, pacritinib, and momelotinib—may not address all the unmet needs in patients with MF, especially in the second-line setting and the setting of disease modification [17,18,19,20]. Multiple “non-JAK inhibitor” molecules are being tested in phase 2 and 3 studies, either as monotherapy or in combination with JAK2 inhibitors [20,21]. In this review, the role of LSD1/KDM1A inhibition as a potential disease-modification strategy in patients with myelofibrosis is appraised.

## 2. Mutations in Epigenetic Regulators in Myelofibrosis

**Figure 1:**
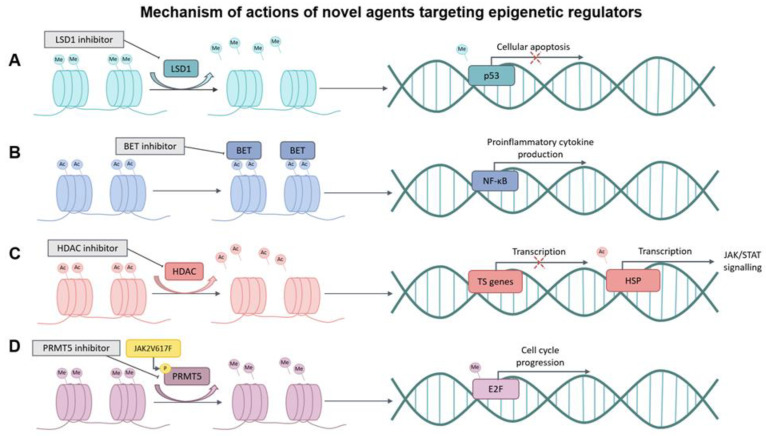
Mechanism of actions of novel agents targeting epigenetic regulators. (**A**) Removal of methyl groups by LSD1, inhibiting p53 methylation and abrogating cellular apoptosis. LSD1 inhibitor antagonizes LSD1 to restore tumor-suppressive effects of p53. (**B**) Anchoring of BET proteins to acetylated lysine residues to activate NF-κB pathway. BET inhibitors block BET proteins and the proinflammatory pathway to reduce synthesis of proinflammatory cytokines. (**C**) Removal of acetyl groups by HDAC, decreasing tumor-suppressor gene transcription while deacetylating HSP to aggravate JAK/STAT signaling pathway. This effect can be overcome by HDAC inhibitors. (**D**) Aberrant phosphorylation of PRMT5 by JAK2V617F, leading to impaired methylation activity. Thus, E2F is methylation for cell cycle progression and myeloproliferation. The aberrant activation can be inhibited by an PRMT5 inhibitor. Me: methylation; Ac: acetylation; LSD1: lysine-specific demethylase-1; BET: bromodomain and extraterminal domain; NF-κB: nuclear factor kappa-light-chain enhancer of activated B cells; HDAC: histone deacetylase; TS genes: tumor suppressor genes; HSP: heat shock protein; JAK: Janus kinase; STAT: signal transducer and activator of transcription; PRMT5: protein arginine methyltransferase 5; E2F: E2F transcription factor 1.

With the advent of next-generation sequencing and whole-genome analyses in myeloid malignancies, mutations in DNA methylation genes (*TET2*, *DNMT3A*, *IDH1/2*), histone modification genes (*EZH2*, *ASXL1*), RNA splicing factors (*SRSF2*, *SF3B1*, *U2AF1*, *ZRSR2*), and transcription factors (*TP53*, *CUX1*, *IKZF1*, *ETV6*, *RUNX1*) have been described in MPNs [22,23,24]. Nevertheless, these mutations are not restricted to MPNs, and are also seen in myelodysplastic syndrome (MDS), AML, and other myeloid malignancies. These mutations are involved in the phenotypic and disease evolution of MPNs. *TET2* (10–20% of MPNs) and *DNMT3A* (5–10% of MPNs) have been found to precede *JAK2*^V617F^ mutations, and have a central role in self-renewal and disease initiation in MPN hematopoietic stem cells (HSCs) [25,26,27,28,29,30,31,32]. The role of *DNMT3A* and *TET2* in the progression to MF or secondary AML is yet to be elucidated. *EZH2* mutations are seen in 5% to 10% of PMF and portend a poor prognosis [33]. In murine MPN models, *Ezh2* loss modifies phenotype and is associated with disease progression [34,35,36]. Mutations in *ASXL* are seen in 25% of PMF and are associated with worse outcomes [37,38,39]. Loss-of-function mutations in *ASXL1* are associated with a higher risk of leukemic transformation. In murine models, *Asxl1* loss is associated with dysplasia, cytopenia, and defective HSC self-renewal [40,41]. *SRSF2* mutations are mostly restricted to ET and PMF and associated with poor outcomes [42]. They are especially enriched in patients with secondary AML from preceding MF. *TP53* mutations are uncommon in PMF or other MPNs in the chronic phase. However, they are found in up to 50% of patients with secondary AML and are often associated with *ASXL1*, *SRSF2*, *IDH1/2*, *CBL*, and *LNK* mutations [43,44]. They are especially common in secondary AML from preceding post-PV or post-ET MF and are associated with *DNMT3A* mutations. Other mutations associated with late events in the clonal progression of MPNs and secondary AML include *RUNX1*, *FLT3*-ITD, *NRAS*, *NF1*, *IKZF1* and *CUX1* [45,46]. Mutations in epigenetic regulators and transcription factors are often associated with advanced MF and increased risk of progression [27,28,47,48,49]. Manipulation of these genetic alterations may offer an additional therapeutic option in patients with an otherwise dismal outcome, and targeting epigenetic regulators with novel agents may potentially alter disease biology in MF (Figure 1).

## 3. The Functional Role of LSD1 in Hematopoiesis

*LSD1* is an essential gene, the loss of which leads to early embryonic lethality [50,51]. The protein also regulates the balance between self-renewal and proliferation [52]. Conditional in vivo *LSD1* knockdown using a doxycycline-inducible short hairpin *LSD1* (*shLSD1*) established LSD1 as a central regulator of HSPCs [53]. An inducible *LSD1* knockdown resulted in profound but reversible thrombocytopenia, neutropenia, and anemia with concurrent monocytosis. *LSD1* knockdown for 27 days led to an increase in circulating multipotent progenitors and HSCs with a concomitant downregulation of chemokine (C-X-C motif) receptor 4 (CXCR4) without affecting the size of the quiescent long-term hematopoietic stem cell (HSC) pool [53].

LSD1 is a key regulator of the progression from pluripotency to terminal differentiation and balancing self-renewal and proliferation [52,54]. LSD1 is recruited to promoters and enhancers of genes essential for normal development by the transcription factors octamer-binding transcription factor 4 (OCT4), SRY (sex determining region Y)-box 2 (SOX2), Nanog, and the coactivator mediator. LSD1 maintains the pluripotency program allowing embryonic stem cells (ESCs) to differentiate. LSD1 is also essential for the complete shutdown of ESC gene expression, as cells undergo transition to more differentiated cell states [54]. LSD1 plays a similar role during myelopoiesis, allowing commitment of progenitors to specific myeloid lineages [55]. Enhancers essential for terminal myeloid differentiation in lineage-specific progenitor cells are activated by the placement of H3K4me1 marks. As progenitors commit to differentiation, LSD1 is significantly downregulated, allowing enhancers and promoters to be gradually activated with progressive addition of methyl or acetyl groups to H3K4 and H3K27, respectively [55].

## 4. LSD1 as an Epigenetic Regulator

Lysine methyltransferases and demethylases are able to catalyze N-methylation and N-demthylation of histone (H) lysines (K) [56,57]. LSD1, or KDM1A, is an enzyme that removes mono- and dimethyl groups from the histone H3 at the critical lysines K4 and K9 [58]. Methylation of histone H3K4 and H3K9 is a posttranslational modification that results in conformational change of chromatins [59,60]. Chromatins are a constellation of nuclear macromolecules and comprise DNA, protein scaffolding, and enzymes that drive RNA transcription and synthesis [61]. The DNA and its protein scaffold of histones form the nucleosome. Each nucleosome comprises two copies of each of the four histone proteins—H2A, H2B, H3, and H4—forming an octamer around which DNA is wrapped. The rates of gene transcription are heavily influenced by the accessibility of transcription factors and RNA polymerase complexes to template DNA at promoters and enhancers [59,60].

Histone and nucleic acid modifications provide binding sites for proteins and components of the transcriptional machinery that affect transcriptional gene silencing or activation. Histone modifications include acetylation (Ac), methylation (Me), phosphorylation (Ph), and ubiquitination (Ub). LSD1 acts as an epigenetic regulator of gene expression by altering the local state of the chromatin. Inhibition of LSD1 results in alteration of gene expression and inhibits the maturation of JAK-STAT activated megakaryocytes and myeloid cells from their progenitors. This also results in the inhibition of self-renewal potential of HSPCs harboring the pathogenic driver mutations [62,63,64].

LSD also regulates nonhistone substrates [65]. LSD1 is localized to specific sites in the genome through various transcription factors that bind DNA [54,66]. Transcription activators, such as V-Myb avian myeloblastosis viral oncogene homolog (MYB) and steroid hormone receptors, as well as repressors, such as growth factor independence 1 transcription repressor (GFI1) and RE-1 silencing transcription factor (REST), recruit LSD1 to specific locations on the genome [67,68]. LSD1 is part of a larger protein complex, containing Co-RE-1 silencing transcription factor (CoREST), nucleosome remodeling and histone deacetylase (NuRD), or other factors that determine cell- and site-specific chromatin remodeling [51,69]. These complexes may also include DNA methyltransferase 1 (DNMT1) and histone deacetylases 1, 2, and 3 (HDAC1, 2, and 3) activities, all of which contribute to maintaining or modifying the epigenetic state at that genomic site [70,71]. Therefore, an important property of LSD1 is its function as a scaffold for other proteins and epigenetic enzymes that are corecruited to genomic sites. LSD1 bound to specific sites precludes the binding of other factors that may influence transcription.

## 5. The Biological Role of LSD1 and the Effect of LSD1 Inhibitors in Murine Models of MPNs

Overexpression of *LSD1* messenger RNA (mRNA) and excess LSD1 protein have been observed in various malignancies, including neuroblastoma, squamous-cell carcinoma, Ewing sarcoma, AML, neuroendocrine tumors, breast cancer, prostate cancer, bladder cancer, small-cell lung cancer, and colorectal cancer [63,64,67,72,73,74]. In MPNs, LSD1 is overexpressed, mainly in the megakaryocytes, erythroid precursors, and to a certain extent in in the early myeloid series [75]. Treatment of various malignant cell types in vitro with LSD1 inhibitors suppresses tumor growth, reduces their invasiveness, reduces clonogenic potential, eliminates cancer stem cells, induces markers of differentiation appropriate to the cell lineage, and induces apoptosis [76,77,78]. In various models of mouse leukemia, treatment with LSD1 inhibitors induced monocytic markers of differentiation, reduced clonogenic potential of leukemia-initiating cells (LICs), and induced apoptosis [78]. LSD1 is expressed in a high proportion of leukemic myeloblasts [79,80]. LSD1 gene expression is among the highest in the malignant myeloid stem and progenitor cell population [77,78]. LSD1 plays a direct role in regulating pathogenic JAK-STAT signaling pathways. The key MPN driver genes *JAK2*^V617F^, *CALR*, and *MPL* activate JAK-STAT signaling via phosphorylation of STAT3, STAT5, and transcription factors, which activate specific genes with pleiotropic effects [81]. STAT3 activity is modulated by methylation on lysine (K140) and is one of the substrates for LSD1 [82]. Proof-of-concept studies have been performed on well-established, preclinical mouse models of MPNs (*JAK2*^V617F^, *Mpl*^W515L^). LSD1 inhibition in *Mpl*^W515L^ mice markedly suppressed myeloproliferation, reducing leukocyte and platelet counts. Spleens in animals treated with LSDi showed a dose-proportional decrease in weight. Histologically, a marked reduction in myeloid proliferation was demonstrated in the bone marrow and the spleen alongside a reversal of extramedullary hematopoiesis (EMH). Intriguingly, there was marked reduction in the degree of bone marrow reticulin fibrosis with LSD1 inhibition. LSD1 inhibition also significantly reduced serum inflammatory cytokine concentrations, in particular the plasma concentration of the chemokine (C-X-C motif) ligand 5 (Cxcl5 or IL-8 in humans), a key mediator of the inflammatory state seen in MPNs. In these mouse models, a reduction in mutant allele burden of driver mutations was also demonstrated. The observation of reduction in the degree of marrow fibrosis and the mutant allele burden of driver genes support the proposition that targeting LSD1 may induce disease modification in MPNs.

## 6. Clinical Data of LSD1 Inhibitors in Myelofibrosis

Given the important roles that LSD1 plays in carcinogenesis, various LSD1 inhibitors have been evaluated in clinical trials. Some of the reported LSD1 inhibitors include tranylcypromine (TCP or PCPA), ORY-1001 (iadademstat), GSK-2879552, IMG-7289 (bomedemstat), INCB059872, CC-90011, and ORY-2001 (vafidemstat). Bomedemstat (IMG-7289, Imago Biosciences, San Francisco, CA) is the most extensively evaluated LSD1 inhibitor in myeloproliferative neoplasms. In *JAK2*^V617F^-postive MPN mice, daily dosing improved blood counts, reduced spleen volumes, reduced marrow reticulin fibrosis, and reduced mutant allele frequencies [83].

Bomedemstat is the only LSD1 inhibitor clinically evaluated in patients with advanced myelofibrosis. In an ongoing phase 2 study in 89 patients with advanced MF, the efficacy and safety of bomedemstat was confirmed [84,85]. Eighty-three percent of the patients had a history of treatment failure with ruxolitinib, with 70% also receiving a second treatment with an unfavorable experience. Thirty-seven percent of patients had received at least one red cell transfusion prior to dosing. The most frequently reported adverse event was thrombocytopenia, an expected observation, given that dosing to grade 3 thrombocytopenia was allowed. The commonest nonhematological toxicity was dysgeusia, reported in 33%, with one discontinuation. Other serious adverse events (SAEs) were reported in 49% of the patients. The most common SAEs, regardless of causality, were cellulitis, diverticulitis, and pneumonia. There were no deaths related to the study drug. The efficacy endpoints were reduction in spleen volume and reduction in total symptom scores (TSS) using the MPN symptom assessment form (SAF). At 24 weeks, 64% of patients had a decrease in spleen volume, with 6% of patients having more than 35% reduction. In patients with a high symptom burden (TSS > 20), 65% had a decrease in TSS, and for 19% of patients, the decrease in TSS was greater than 50%. By week 12, 44% of patients had an increase in hemoglobin of 1 g/dL or more and 46% had stable hemoglobin. At the data cutoff point, of 21 patients, three had become transfusion-independent. The improvement in hemoglobin is an intriguing observation and is likely to address the issue of anemia associated with ruxolitinib. A possible explanation for this observation is that the modulation of transcription by LSD1 is lineage-specific or that expression of the γ-globin gene is altered with LSD1 inhibition [86,87]. Eighty-five percent of patients had stable or improved fibrosis score of at least one grade. Patients with an elevated inflammatory chemokine, such as chemokine ligand 5 (CCL5), had a measurable decrease into normal concentrations. The mutant allele frequencies (MAF) of driver and nondriver mutations among the 261 genes were serially sequenced. Fifty-two percent of patients had a decrease in MAF, with ASXL1 being the most sensitive to bomedemstat. No patient progressed to secondary AML in this study.

## 7. Conclusions and Future Perspectives

As we progress to the era of novel therapies that could alter disease biology, there is a need to utilize end points that inform us how these novel agents could alter the disease trajectory (Table 1). The current data available from clinical studies support the definition of disease modification that comprises clinically meaningful improvement in survival, reduction in the risk of leukemic transformation, restoration of normal hematopoiesis, significant reduction in bone marrow fibrosis, and reduction in the clonal burden of the disease. Achieving disease modification will ultimately lead to beneficial effects in traditional outcome measures, such as symptom improvement and control of splenomegaly. In spite of the limited disease-modifying effect of JAK inhibitors, the JAK-STAT pathway remains pivotal. This is supported by the modest effect of single-agent bomedemstat on spleen volume. Treatment strategies combining JAK inhibitors and novel agents, such as bomedemstat, will provide synergic effects in improving outcomes and altering disease biology. The selection of JAK inhibitors to be used with bomedemstat should take into account the disease characteristics and clinical needs, such as the presence of anemia (where fedratinib or momelotinib could be considered) or thrombocytopenia (where pacritinib could be considered). Based on the effect of LSD1 inhibition on erythropoiesis, it will also be intriguing to observe if bomedemstat can circumvent anemia caused by ruxolitinib. In addition, the impressive responses seen with bomedemstat in patients with essential thrombocythemia [88] suggest that LSD1 inhibitors could potentially be beneficial when used earlier in MF, such as in prefibrotic or early PMF, an area that is yet to be explored.

## Figures and Tables

**Table 1:** Selected novel agents showing improvement in surrogate markers for disease modification in myelofibrosis, either as single agent or in combination with ruxolitinib.

Class	Drug	Study Population	Design	SVR35 at 24 Weeks	TSS50 at 24 Weeks	Anemia Response	VAF Reduction	BM Fibrosis Reduction
LSD1 inhibitor	Bomedemstat [84,85]	Ruxolitinib exposed: 83% (74/89) N = 89	Phase 2 (ongoing) Single-agent bomedemstat	6% (3/50)	19% (5/26)	In TD patients: 52% (11/21) had stable or reduced transfusion burden; 14% (3/21) became TI	VAF reduction 52% (36/69), most frequently in *JAK2*^V617F^ and/or *ASXL1*	31% (16/52) improved by 1 grade 50% (26/52) stable
BET inhibitor	Pelabresib (MANIFEST) [89,90]	Both JAKi exposed and JAKi naïve, N = 271	Phase 1/2 (ongoing) Arm 1: JAKi exposed (pelabresib) Arm 2: JAKi exposed (pelabrasib + ruxolitinib) Arm 3: JAKi naïve (pelabresib + ruxolitinib)	Arm 1: 11% (7/64) Arm 2: 20% (16/81) Arm 3: 68% (57/84)	Arm 1: 28% (18/64) Arm 2: 37% (30/81) Arm 3: 56% (46/82)	Arm 1: In TD patients, 16% (4/25) became TI Arm 2: In TD patients, 36% (13/36) became TI Arm 3: In patients with Hb < 10 g/dL; Hb improved by 1 g/dL	Not reported	Arm 1: 23% (7/30) improved at 24 weeks Arm 2: 25% (9/36) improved at 24 weeks Arm 3: 31% (16/52) improved at 24 weeks
Telomerase inhibitor	Imetelstat (IMBark) [91]	JAKi exposed N = 59	Phase 2 (complete) Single-agent imetelstat	10.2% (6/59)	32.2% (19/59)	In TD patients, 25% (3/12) became transfusion-independent	42% had ≥25% reduction in VAF	41% (15/37) had reduction in BM fibrosis
BH3 mimetic; Bcl-2/Bcl-X_L_ inhibitor	Navitoclax (REFINE) [92]	Ruxolitinib exposed N = 174	Phase 2 (ongoing) Navitoclax +/− ruxolitinib	27% (9/34)	30% (9/34)	In TD patients or patients with Hb < 10 g/dL; TI or ≥ 2 g/dL in 64% (7/11)	46% (12/26) had >10% reduction in VAF	21% (7/34) had BM fibrosis reduction at 24 weeks
MDM2 inhibitor	Navtemadlin (BOREAS) [93]	JAKi exposed N = 113	Phase 2 (ongoing) Single-agent navtemadlin	Not reported	Not reported	Not reported	34% had ≥20% reduction in VAF	27% ≥ 1 grade reduction in BM fibrosis
Hypomethylating agent	Azacitidine [94]	JAKi naïve N = 60	Phase 2 Ruxolitinib + azacitidine	NR	54% (25/46)	In TD patients, 20% (1/5) became TI	81% (13/16) had reduction in *JAK2*^V617F^ VAF at 24 weeks	57% (8/14) had reduction in BM fibrosis at 24 weeks

SVR35: ≥35% reduction in spleen volume from baseline to 24 weeks; TSS50: ≥50% reduction in total symptom score from baseline to 24 weeks; VAF: variant allele frequency; BM: bone marrow; LSD1: lysine-specific demethylase 1; BET: bromodomain and extraterminal; Bcl-2: B-cell lymphoma 2; MDM2: murine double minute 2; TD: transfusion-dependent; TI: transfusion-independent; JAKi: JAK inhibitor; Hb: hemoglobin; NR: not reported.

## Data Availability

Not applicable.
