# Correction: Gill, H. Lysine-Specific Demethylase 1 (LSD1/KDM1A) Inhibition as a Target for Disease Modification in Myelofibrosis. *Cells* 2022, *11*, 2107

**DOI:** 10.3390/cells11244126

**Published:** 2022-12-19

**Authors:** Harinder Gill

**Affiliations:** Department of Medicine, LKS Faculty of Medicine, School of Clinical Medicine, The University of Hong Kong, Hong Kong SAR, China; gillhsh@hku.hk; Tel.: +852-22554542

## Text Correction

The authors would like to make the following corrections to the published paper [1]:

The acknowledgments are missing. Bomedemstat, the most successful LSD1 inhibitor, was developed by Dr. Hugh Y Rienhoff and his team at Imago Biosciences, and many ideas in this review were inspired by Dr. Rienhoff and his story. Therefore, the author feels a strong need to acknowledge them in a correction notice. The corrected acknowledgments should read as follows: 

**Acknowledgments:** The author would like to acknowledge Hugh Young Rienhoff and his team at Imago Biosciences for their work on the role of LSD1 and the development of IMG-7289 (Bomedemstat), which inspired this manuscript.

The authors state that the scientific conclusions are unaffected. This correction was approved by the Academic Editor. The original publication has also been updated.
